# cNap1 bridges centriole contact sites to maintain centrosome cohesion

**DOI:** 10.1371/journal.pbio.3001854

**Published:** 2022-10-25

**Authors:** Robert Mahen

**Affiliations:** 1 The Medical Research Council Cancer Unit, University of Cambridge, Hills Road, Cambridge, United Kingdom; 2 Photonics Group, Department of Physics, Imperial College London, London, United Kingdom; Institut Curie, FRANCE

## Abstract

Centrioles are non-membrane-bound organelles that participate in fundamental cellular processes through their ability to form physical contacts with other structures. During interphase, two mature centrioles can associate to form a single centrosome—a phenomenon known as centrosome cohesion. Centrosome cohesion is important for processes such as cell migration, and yet how it is maintained is unclear. Current models indicate that pericentriolar fibres termed rootlets, also known as the centrosome linker, entangle to maintain centriole proximity. Here, I uncover a centriole–centriole contact site and mechanism of centrosome cohesion based on coalescence of the proximal centriole component cNap1. Using live-cell imaging of endogenously tagged cNap1, I show that proximal centrioles form dynamic contacts in response to physical force from the cytoskeleton. Expansion microscopy reveals that cNap1 bridges between these contact sites, physically linking proximal centrioles on the nanoscale. Fluorescence correlation spectroscopy (FCS)-calibrated imaging shows that cNap1 accumulates at nearly micromolar concentrations on proximal centrioles, corresponding to a few hundred protein copy numbers. When ectopically tethered to organelles such as lysosomes, cNap1 forms viscous and cohesive assemblies that promote organelle spatial proximity. These results suggest a mechanism of centrosome cohesion by cNap1 at the proximal centriole and illustrate how a non-membrane-bound organelle forms organelle contact sites.

## Abstract 2

During interphase, two mature centrioles can associate to form a single centrosome; this "centrosome cohesion" is important for processes such as cell migration, but how is it maintained? This study combines live cell quantitative imaging, expansion microscopy and ectopic tethering to provide insights into the mechanisms by which centrioles maintain spatial proximity inside human cells.

## Introduction

Organelle contact sites are critical to diverse cellular functions. Membrane-bound organelles associate via dedicated molecular complexes that perform functions such as membrane tethering [1]. How non-membrane-bound molecular assemblies form physical contacts with other cellular structures is less clear.

Centrosomes are microtubule-organising centres that mediate fundamental cellular processes including cell division, polarity, and motility. Centrosomes exist in the cellular interior without a bounding membrane, dynamically interacting with structures such as the cell membrane and mitotic spindle [2]. During interphase, mammalian centrosomes contain 2 mature microtubule-based structures called centrioles. Centrioles associate together in a process termed centrosome cohesion [3,4]. Centrosome cohesion is important for mitosis, ciliary function, and cell migration [5–8], and thus understanding its molecular and biophysical basis is an important question.

Rootlets, also known as the centrosome linker, are pericentriolar fibres found at centrioles [3,9,10]. Across the Animalia kingdom, rootletin/CROCC protein (*CROCC*) is a key component of rootlets [5,11–14]. Rootlets are frequently prominent when centrioles form cilia in specialised cell types such as mechanosensory neurons, and may form links between centrioles as part of polarised multiciliary arrays [10]. Rootletin loss of function studies have demonstrated that rootlets are required for centrosome cohesion in non-ciliated human cells [3,15,16]. One model postulates that rootlets maintain centrosome cohesion by entangling together, therefore establishing direct physical links between centrioles [3,9,17–19]. These links are likely weak or dynamic, since centrioles can transiently separate, potentially in response to physical force from the cytoskeleton [20–24].

cNap1 (also known as CEP250) is a rootletin paralog found at the proximal centriole, spatially adjacent to rootlet fibres [18,19,25,26]. Truncating mutations in cNap1 have been suggested to cause mammalian retinal and developmental dysfunction [7,27,28]. cNap1 binds to rootletin in biochemical assays, dissociates from centrosomes when they split during mitosis, and is required for rootlet formation at centrosomes, suggesting that it anchors rootlets to centrioles [3,9,15,26,29]. cNap1 disruption also causes loss of centrosome cohesion [7,15,29,30]—a phenomenon attributed to rootlet disruption rather than a more direct role in centrosome cohesion. Little is known about the biophysical properties of cNap1 that allow it to maintain centrosome cohesion or the molecular basis of centriole–centriole contact sites.

Here, by studying the biophysical properties and nanoscale architecture of cNap1 at centriole–centriole contact sites, I discover that it directly maintains centrosome cohesion. Live-cell imaging and expansion microscopy of endogenous cNap1 shows that it bridges between dynamic centriole–centriole interfaces. cNap1 accumulates at micromolar concentrations, forming supramolecular assemblies with viscous material properties that cohesively maintain organelle spatial proximity. I propose a model of centrosome cohesion explaining how organelle solidity is balanced against organelle plasticity using dynamic cNap1 assemblies.

## Results

### Proximal centriole pairs and rootlets form dynamic contacts during centrosome cohesion

To simultaneously track the spatiotemporal behaviour of both proximal centrioles and rootlets in living cells, genome editing was used to create U2OS cells expressing endogenously tagged fluorescent cNap1 and rootletin (cNap1-mScarlet-I and rootletin-meGFP). Cell lines were carefully validated; precise genomic tagging was ensured by a combination of overlapping genomic PCR and imaging (**S1A–S1C Fig** and **Materials** and **methods**). Clones were screened to identify cells with all cNap1 alleles homozygously modified. cNap1-mScarlet-I and rootletin-meGFP functionality was ensured by measuring centrosome cohesion, which was indistinguishable in genome-edited and wild-type cells (**S1D Fig**.). These considerations confirmed that cNap1 and rootletin were tagged at functional, endogenous levels.

**Fig 1:**
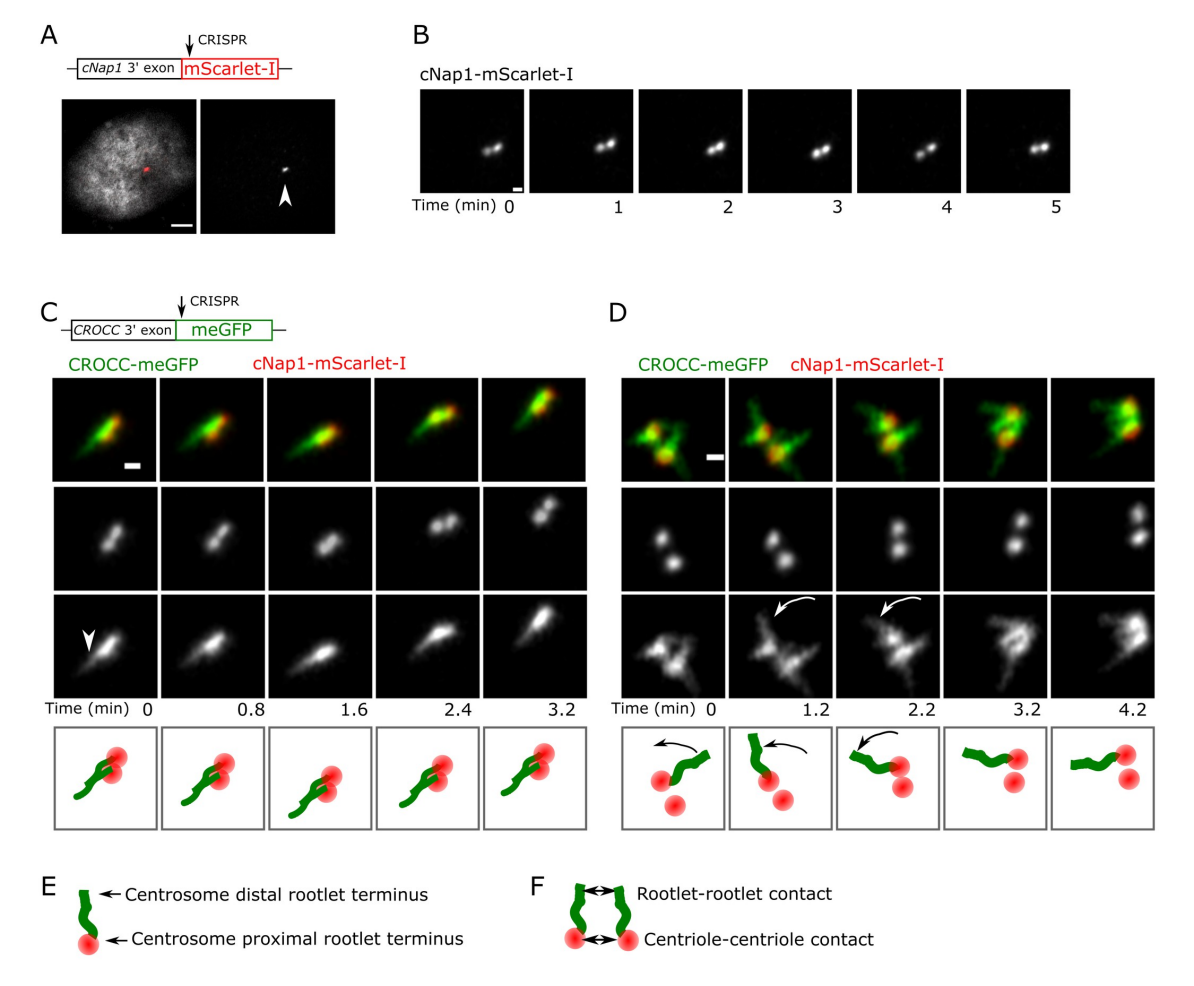
Proximal centriole pairs and rootlets form dynamic contacts during centrosome cohesion. **(A)** Endogenously tagged cNap1-mScarlet-I localises to regions of high concentration at proximal centrioles (denoted by the arrow). A merged image showing both cNap1-mScarlet-I and the nucleus is shown on the left panel, the right panel shows only cNap1-mScarlet-I. The nucleus is labelled with Hoechst 33342. Scale: 3 μm. **(B)** Time-lapse imaging of endogenously tagged cNap1-mScarlet-I at 1-min intervals shows dynamic contacts. The images show a single z-slice from 3D data. Scale: 0.5 μm. (**C and D**) Time-lapse 2-colour Airyscan imaging of endogenously tagged cNap1-mScarlet-I and rootletin-meGFP. Scale: 0.5 μm. The arrowhead in (**C**) denotes a potential point of contact between rootlets from different centrioles. The arrows in **(D**) denote independent movement of a rootlet distal terminus relative to other rootlets. (**E**) Cartoon depiction of the arrangement of cNap1 and rootletin at centrosomes; centrosome proximal cNap1-mScarlet-I is attached to rootlet termini. (**F**) Cartoon depiction of either rootlet–rootlet or centriole–centriole contact sites. Data underlying this figure can be found in S1–S3 Movies.

I reasoned that if centrosome cohesion is mediated by direct links between centrioles, these contact points might be visible with imaging. cNap1-mScarlet-I enriched strongly at centrosomes relative the surrounding cytosolic pool, forming either 1 or 2 foci corresponding to the proximal centrioles (**Figs 1A** and **S1E**), as expected from prior electron microscopy [26]. Live-cell Airyscan time-lapse imaging showed that on the seconds to minutes timescale, the relative spatial proximity of centriole pairs was variable (**Fig 1B** and **S1F and S1G** and **S1 Movie**). Proximal centrioles thus transiently formed contacts as they collided together during continuous movement. Rootlet fibres were also mobile during centriolar movements. Rootlets from both centrioles could either form contacts, or alternatively move independently, apparently not in contact (**Fig 1C** and **1D and S2 and S3 Movies**). Throughout these dynamic movements, cNap1-mScarlet-I foci were always present at the centrosome-proximal termini of rootletin-meGFP fibres (**Fig 1E**). Thus, simultaneous imaging of endogenously tagged cNap1 and rootletin reveals that both proximal centrioles and rootlets form dynamic contacts during centrosome cohesion (**Fig 1F**).

### Endogenous cNap1 bridges proximal centrioles at the nanoscale

To investigate centriole–centriole contact sites below the diffraction limit of light microscopy, I turned to ultra-expansion microscopy (U-ExM) [31]. U-ExM increases resolution by expanding fixed cells approximately 4-fold in size, therefore achieving resolution on the tens of nanometres scale [31]. Since centriole diameter and length have previously been determined, I used anti-acetylated tubulin staining of centrioles to ensure isotropic and efficient expansion in my experimental setup, with previously described protocols optimised to maintain centriole morphology [31,32]. Anti-acetylated tubulin staining of centriolar barrels gave a diameter of approximately 190 nm, consistent with previous work, and demonstrating isotropic and efficient expansion [32].

**Fig 2:**
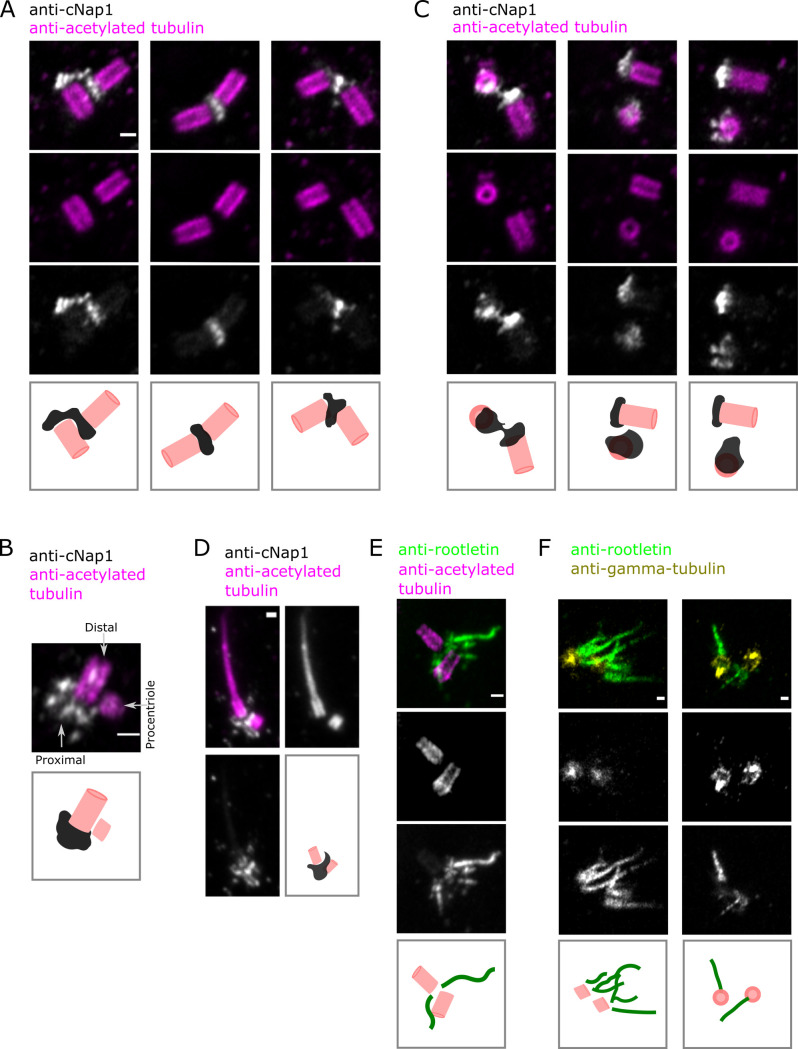
Endogenous cNap1 bridges proximal centrioles at the nanoscale. (**A–C**) U-ExM of centrioles labelled with anti-acetylated tubulin antibody (pink) and cNap1 labelled with anti-cNap1 antibody (grey). The images show single Airyscan z-slices. Cartoons depict simplified centriole and cNap1 orientations. (**D**) ExM of centrioles labelled with anti-acetylated tubulin antibody (pink) and cNap1 labelled with anti-cNap1 antibody (grey) in a ciliated HPNE cell. The image shows a maximum intensity projection from 3D data. The cartoon depicts only the ciliary base region for simplicity. (**E**) U-ExM of rootlets stained with anti-rootletin antibody (green) and centrioles stained with anti-acetylated tubulin antibody (pink). (**F**) U-ExM of rootlets stained with anti-rootletin antibody (green) and centrioles stained with gamma-tubulin antibody (yellow). Single z-slices are shown. Panels (A), (C), (E), (F) show wild-type U2OS, (B) is endogenously tagged cNap1-mScarlet-I U2OS and (D) shows hTERT-HPNE cells. Across all panels, the scale is 200 nm, and each column represents a different cell. Data underlying this figure can be found in S2 Fig. U-ExM, ultra-expansion microscopy.

Consistent with the live-cell imaging data (**Fig 1**), centriole pairs occupied variable orientations relative to each other in populations of U2OS cells (**Fig 2A)**. U-ExM staining of cNap1 with a small interfering RNA (siRNA)-validated antibody showed that it accumulates at the proximal centriole (**Figs 2B** and **S2A**) [26]. Strikingly, cNap1 formed structures that bridged both centrioles in 39% of interphase cells (from a total of 80 cells imaged). Thus, cNap1 from both centrioles either coalesced together into a single ellipsoid-shaped structure or 2 ellipsoids could form junctions between 2 centrioles in various orientations. In the remaining 61% of cells, cNap1 formed 2 unconnected spatially separate structures. Imaging end-on down the centriole barrel highlighted variability in the cNap1 structures as asymmetrical ellipsoids (**Fig 2C**). A montage of approximately 60 different cells is presented in **S2B Fig** to document this variability in cNap1 orientation and shape. cNap1 bridging between centrioles was also observed in a different cell type, hTERT-HPNE (**S2C Fig**), both in ciliated and non-ciliated cells (**Fig 2D**).

U-ExM of rootletin showed that it too was present in multiple orientations, also consistent with the live-cell imaging data (**Fig**s **2E** and **S2D**). With centrioles adjacent, rootlets could either be oriented radially into the cytoplasm without overlap, or formed contact points with the rootlets from the adjacent centriole (**Fig 2F**). Together, these results demonstrate that cNap1 can either form separate assemblies on each proximal centriole or can bridge centriole pairs as a contiguous structure.

### cNap1 forms viscous condensates that coalesce

**Fig 3:**
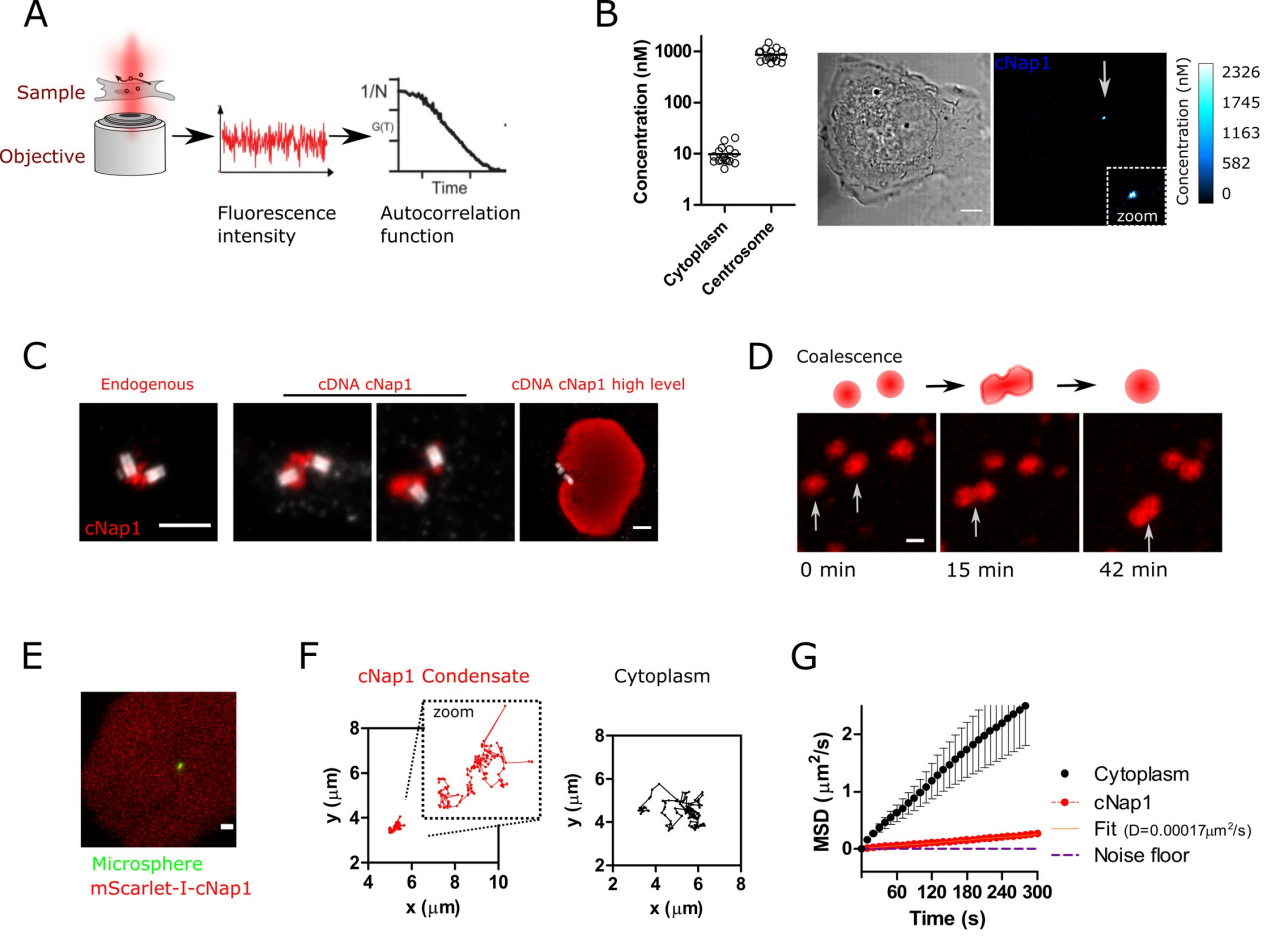
cNap1 forms viscous condensates. (**A**) Schematic of FCS measurements. Fluorescence fluctuations within a confocal volume are converted into absolute concentrations using the FCS autocorrelation function and measurements of the confocal volume size. Image intensities are converted from arbitrary fluorescence intensities into concentration maps. (**B**) FCS-calibrated imaging of homozygously tagged endogenous cNap1-mScarlet-I, either in the cytoplasm or the centrosome of U2OS cells. The representative image shows a single cell, coloured relative to cNap1 concentration. Scale: 5μm. The dot plot horizontal lines show the mean from a population of cells and each dot represents a single cell. (**C**) Comparative U-ExM of endogenous cNap1 and cDNA-expressed cNap1. Each panel shows a different cell and cNap1 is shown in red. Centrioles are marked by anti-acetylated tubulin staining (white). Scale: 1 μm throughout. The panels show different sized areas. (**D**) Live-cell time-lapse imaging of mScarlet-I-cNap1, showing coalescence. Maximum intensity projections are shown. Scale: 1 μm. (**E**) Green fluorescent microsphere (green) embedded within mScarlet-I-cNap1 (red). A single z-slice is shown. Scale: 1 μm. (**F**) Two example trajectories of bead movement when either embedded in cNap1 (left, red) or in the cytoplasm (right, black). (**G**) MSD of microspheres embedded in cNap1 (red) or in the cytoplasm (black). The lines show weighted means (±SEM) from *N* = 51 (transfected, condensate) and *N* = 43 (untransfected, cytoplasm) tracks taken in 3 independent experiments. The orange line shows the fit from which the diffusion coefficient of 0.00017 μm^2^/s was calculated (R^2^ = 0.996). The noise floor is plotted as a dashed purple line, obtained through measurement of immobilised beads with the same imaging conditions. The data underlying the plots can be found in S1 Raw Data. FCS, fluorescence correlation spectroscopy; MSD, mean squared displacement; U-ExM, ultra-expansion microscopy.

I investigated further the biophysical properties of cNap1 that allow it to bridge across centriole pairs. Fluorescence correlation spectroscopy (FCS) calibrated imaging was used to measure endogenous cNap1-mScarlet-I protein copy number in living cells, where FCS measurements on freely diffusing components are used to calibrate fluorescence confocal images into absolute concentration (**Fig 3A** and **Materials and methods**). Centrosomal cNap1 concentrations were high compared to the cytosolic pool, with a mean centriolar concentration of approximately 862 ±59 nm, compared to approximately 10 ±1 nm in the cytosol (mean, SEM) (**Fig 3B**). Using a measure of total cNap1 centriolar size, this concentration corresponds to a mean of approximately 160 cNap1 proteins per centrosome. This is in broad agreement with a previous proteomics-based estimate [33], and shows that many tens of cNap1 proteins amass at the proximal centriole.

Overexpression of mScarlet-I-cNap1 or untagged cNap1 from cDNA expression plasmids resulted in the accumulation of cNap1 both at centrioles and as patches in the cytosol, in agreement with previous work (**S3A and S3B Fig** and **S4 Movie**) [9,34,35]. Cytosolic patches appeared spontaneously only when cytosolic concentration exceeded approximately 100 nm (**S3C Fig**). U-ExM of cDNA-expressed mScarlet-I-cNap1 showed similar morphologies to endogenous protein, differing primarily in size, being larger at higher levels (**Fig 3C**). Time-lapse imaging on a timescale of minutes to hours showed that like endogenous cNap1, overexpressed cytoplasmic and centrosomal mScarlet-I-cNap1 had the capability to coalesce, through the formation of intermediate bridged shapes prior to one contiguous structure (**Fig 3D** and **S5 Movie**). mScarlet-I-cNap1 patches also showed shape changes over time independent of coalescence, suggestive of cohesive properties within each patch (**S6 Movie** and **S3D Fig)**.

Much recent work has focussed on the material properties of non-membrane-bound cellular organelles, hypothesising that they have material properties equivalent to states of matter such as liquids [36,37]. Since cNap1 patches coalesce and flow in a liquid-like fashion by time-lapse imaging, I investigated the viscosity of cytoplasmic mScarlet-I-cNap1 with single particle tracking microrheology of microspheres embedded within them (**Fig 3E**). Bead movement within cNap1 was slowed relative to the cytosol, as seen in a lower relative mean squared displacement (MSD) over time (**Fig 3F** and **3G**). In a log–log plot of MSD, diffusion within a viscous fluid results in a diffusive exponent α of 1 (MSD = 4DT^α^) [38,39]. Microspheres diffusing in cNap1 had a diffusive exponent of 0.93 ± 0.04, consistent with Brownian diffusion (**S3E Fig** and **Materials and methods**). Bead diffusion coefficient within cNap1 was 0.00017 μm^2^/s (25th to 75th percentile 0.0001 to 0.0002), indicating a viscosity of approximately 15.6 Pa-s (25th to 75th percentile 9.8 to 25.2) according to the Stokes–Einstein relation (see **Materials and methods** for details). This is approximately 15,000 times more viscous than water at 21°C. Consistently, fluorescence recovery after photobleaching (FRAP) indicated that cNap1 within condensates had limited turnover over short timescales of up to approximately 30 s (**S3F Fig)** [34]). Taken together, these observations suggest that cNap1 forms viscous supramolecular assemblies or biomolecular condensates that have a propensity to coalesce.

### cNap1 condensate formation promotes rootlet end-binding but not centrosomal localisation

**Fig 4:**
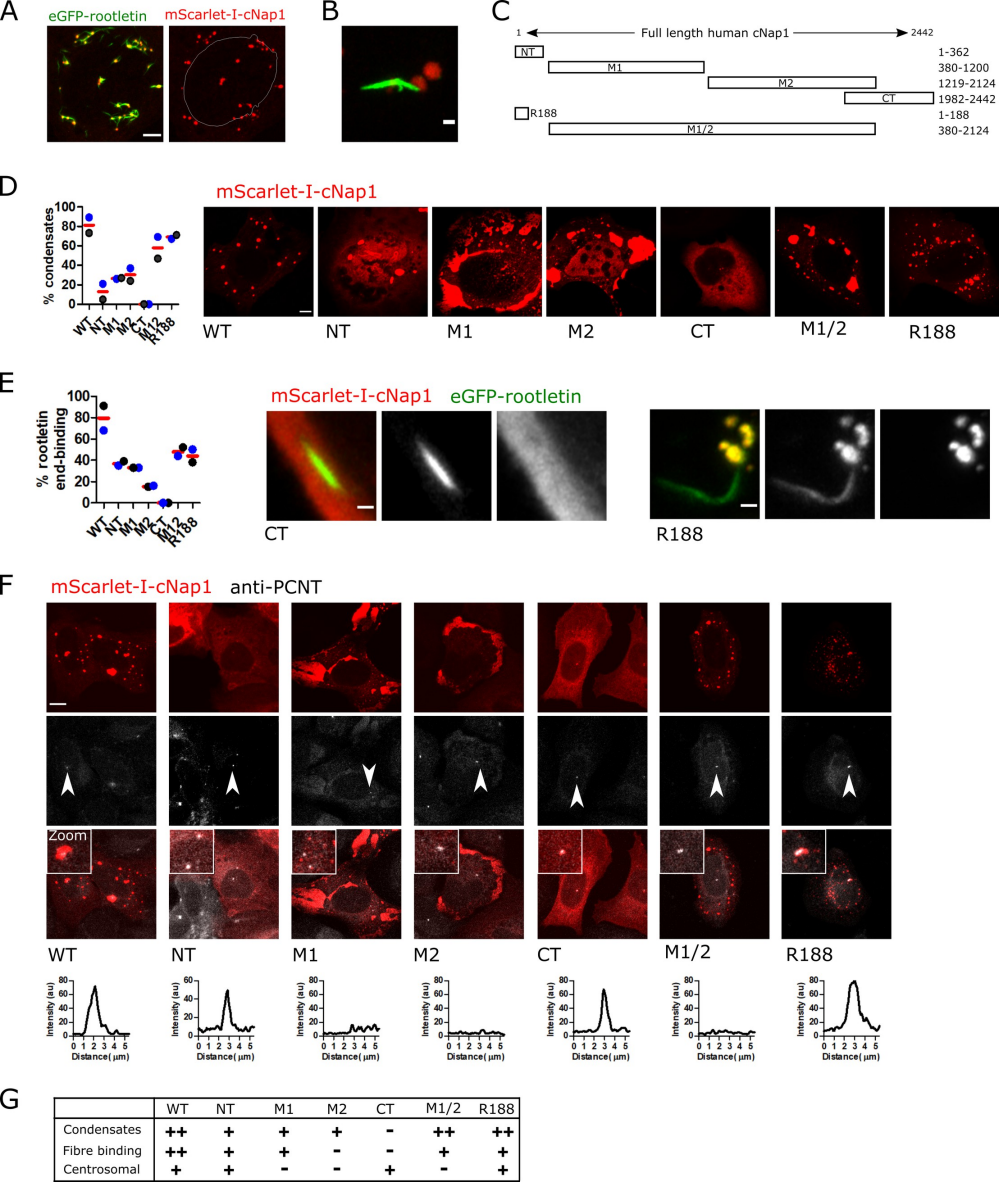
cNap1 condensate formation promotes rootlet end-binding but not centrosomal localisation. (**A**) Co-overexpression of mScarlet-I-cNap1 (red) and eGFP-rootletin (green) in a single cell. Scale: 5 μm. The white line indicates the location of the nucleus for reference. (**B**) Detailed view of cytoplasmic mScarlet-I-cNap1 associated with eGFP-rootletin fibre. Scale bar: 1 μm. (**C**) Schematic representation of cNap1 protein truncations. Numbers denote amino acids from the N-terminus. (**D**) Cytoplasmic condensate formation by cNap1 truncations. Approximately 300 cells were measured in each condition from 2 biological replicates. Each dot depicts the mean percentage of cells containing >1 condensate, colour coded according to replicate. The red horizontal bars show the mean of the experimental repeats. The images show a representative example cell with condensates (except CT, which has none). Scale: 5 μm. (**E**) Rootletin fibre binding by cNap1 truncations. The graph plots the percentage of rootletin fibres associated with cNap1 condensates, where each dot represents the mean from an independent experiment, and the red horizontal line indicates the mean of the experimental repeats. Approximately 300 cells were measured in each condition. Cells without condensates were excluded from the analysis. The images show a representative rootletin fibre (green) with cNap1 CT or R188 (red). Scale: 10 μm. (**F**) Centrosomal localisation of cNap1 truncations. The representative confocal images show mScarlet-I-cNap1 truncations (red) and co-staining of centrosomes with anti-PCNT antibody (white). Centrosomes are indicated by the arrows. Maximum intensity projections are shown. The “smooth” function was used in Fiji and image brightness and contrast are changed for display purposes. The graphs plot a line profile of the intensity of cNap1 across the centrosome in the image. Scale: 10 μm. (**G**) Summary of ectopic mScarlet-I-cNap1 truncation properties, from the experiments in (**D–F**). ++, +, and–denote decreasing amounts, respectively. The data underlying the plots can be found in S1 Raw Data.

cNap1 has previously been shown to bind rootletin by yeast 2-hybrid and coimmunoprecipitation [3,9]. I therefore investigated whether rootletin is recruited into ectopic cytoplasmic mScarlet-I-cNap1 condensates. mScarlet-I-cNap1 was co-expressed in HeLa cells stably expressing eGFP-rootletin, to allow dual-colour time-lapse imaging of the spatiotemporal behaviour of both transgenes during condensate formation. Surprisingly, eGFP-rootletin was not recruited into cNap1 condensates per se. Instead, mScarlet-I-cNap1 condensates bound to eGFP-rootletin fibre termini (**Fig 4A** and **S7 Movie**). Thus, approximately 63% of eGFP-rootletin fibres were coincident with mScarlet-I-cNap1 patches, approximately 18 hours after mScarlet-I-cNap1 transfection in a population of cells. cNap1 predominantly bound to the ends of cytoplasmic rootletin fibres, attaching to either one or both ends (**Fig 4B**). mScarlet-I-cNap1 was not just temporarily colocalised with rootletin fibre termini, but stably attached, since they could translocate together over minutes (**S8 Movie).** Thus, mScarlet-I-cNap1 binds to rootletin fibre termini when overexpressed, forming condensates in the same spatial arrangement as endogenous cNap1 foci at centrosomes (**Fig 1**).

To investigate whether individual cNap1 protein domains are sufficient for condensate formation and rootletin binding, cNap1 was divided into a series of separate fragments [40]. These protein fragments consist of either the N terminus, the C terminus, the middle domain 1, the middle domain 2, or both the middle domains (**Fig 4C**; respectively named here; NT, CT, M1, M2, or M1/2). An R188 mScarlet-I-cNap1 truncation was also created, since it is the site of a truncating mutation (R188) identified in a consanguineous family with retinal impairment [27,28] and close to a nearby truncating reside (169) reported to cause developmental defects in cows [7].

No single cNap1 protein domain was fully sufficient for condensate formation at wild-type levels (**Fig 4D**). Instead, dependent on the domain, 0% to 70% of cells formed cytoplasmic condensates, relative to approximately 80% in wild type. I investigated whether condensate formation influenced cNap1 rootlet-end binding. Rootletin fibre end-binding correlated with condensate formation capability, when analysing only cells that formed condensates. Wild type thus had the highest rootletin fibre end-binding and other truncations had lower levels (**Fig 4E).** Importantly, and in contrast to rootletin fibre end-binding, condensate formation was not essential for centrosomal localisation (**Fig 4F**). Thus, in agreement with previous work [15], either of the terminal domains (NT or CT) were sufficient for centrosomal localisation, as was R188, despite differing condensate-forming ability (**Fig 4G**). These results together suggest that condensate formation promotes cNap1-rootletin fibre end-binding but is not essential for centriolar localisation.

### cNap1 is sufficient for organelle cohesion

**Fig 5:**
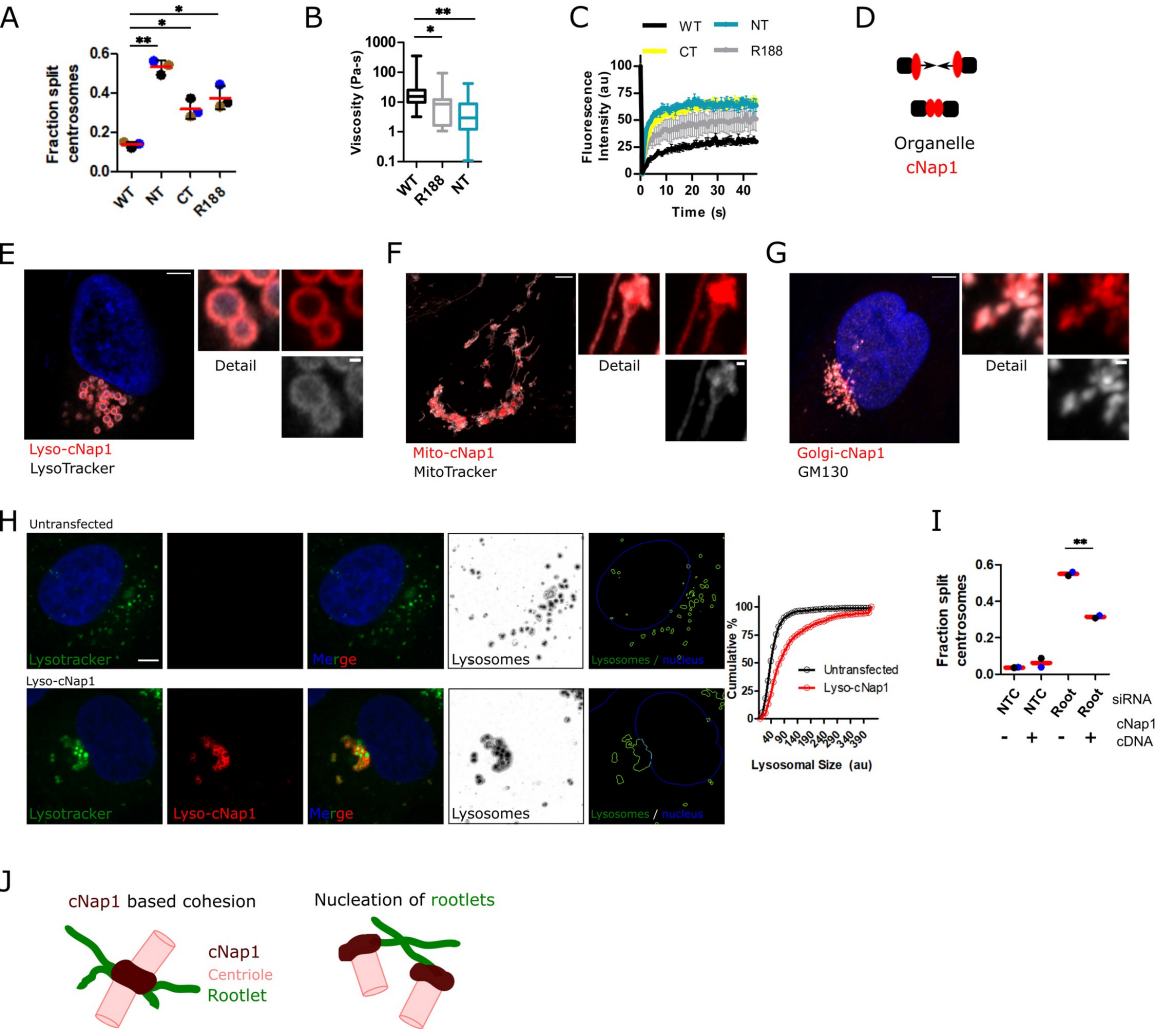
cNap1 is sufficient for organelle cohesion. (**A**) cNap1 truncations NT, CT, and R188 disrupt centrosome cohesion. The bar graph plots the percentage of cells with centrioles separated >1.6 μm, determined from anti-PCNT staining and confocal imaging, in 3 experiments, measuring in a minimum of 83 cells in total per condition. The mean and the standard deviation are shown. Dots represent biological repeats, colour coded in sets. The asterisks denote significant differences by paired *t* test (WT vs. NT *P* = 0.0014, WT vs. CT *P* = 0.0379, WT vs. R188 *P* = 0.0209). (**B**) Viscosity of cNap1 wild type, NT and R188 truncations calculated from the MSD of microsphere movement within them. The box and whiskers plot shows the min to max values and the middle horizontal bars the medians, from 51, 40, and 20 tracks, respectively. (**C**) cNap1 truncations NT, CT, and R188 have an increased rate of FRAP recovery at centrosomes relative to wild type. The graph plots the mean and standard deviation of 3 separate experiments. Approximately 30 cells were measured in each condition. (**D**) Theory that organelle-associated cNap1 promotes organelle spatial proximity. **(E**) Lyso-cNap1 (red) forms spherical structures coating LysoTracker positive vesicles (grey). The image shows an Airyscan confocal z-slice. Scale in large image: 5 μm, scale in detail image: 0.5 μm. (**F**) Mito-cNap1 (red) localises adjacent to mitochondria as marked by MitoTracker (grey). Scale in large image: 5 μm, scale in detail image: 0.5 μm. (**G**) Golgi-cNap1 (red) forms elongated structures adjacent to the Golgi, as shown by co-staining with GM130 (grey). Scale in large image: 5 μm, scale in detail image: 0.5 μm. (**H**) LysoTracker positive vesicle localisation in the presence or absence of lyso-cNap1 (bottom and top panels, respectively). The images show Airyscan z-slices of lyso-cNap1 (red), LysoTracker (green), and DNA Hoechst 33342 (blue). The inverted images (marked “lysosomes”) show automated LysoTracker probability segmentation produced in Ilastik. The images marked “lysosomes and nuclei” show binary segmentation produced in CellProfiler. The cumulative histogram quantitates LysoTracker positive vesicle size either with or without lyso-cNap1 expression in approximately 500 cells. (**I**) Loss of centrosome cohesion caused by rootletin siRNA is partially rescued by mScarlet-I-cNap1. The bar graph plots the percentage of cells with centrioles separated >1.6 μm, determined from anti-PCNT staining and confocal imaging. Each dot is colour coded according to biological replicate and the horizontal bars show the mean (±SD) of the replicates. The asterisk denotes a significant difference by paired *t* test (*P* = 0.0084). (**J**) Model of cNap1-based centrosome cohesion. Centrosomes are formed from 2 mature centrioles that dynamically split and rejoin during interphase. cNap1 accumulates at the proximal end of each centriole, at a concentration of approximately 1 μm and bridges across centriole contact sites, acting as a molecular glue that balances force from the cytoskeleton with cohesion. In parallel, cNap1 promotes rootlet formation or anchoring through binding to rootletin fibre termini. Both rootlets and cNap1 contribute to centrosome cohesion. The data underlying the plots can be found in S1 Raw Data. FRAP, fluorescence recovery after photobleaching; MSD, mean squared displacement; siRNA, small interfering RNA.

Previous loss of function studies have shown that cNap1 is required for centrosome cohesion [7,15,29,30]. In agreement, expression of terminal cNap1 truncations with centrosomal localisation, namely CT, NT, and R188, reduced centrosome cohesion relative to wild-type matched control (**Fig 5A)** [15]. I reasoned that if the material properties of cNap1 contribute to centrosome cohesion, then mutants with loss of centrosome cohesion might have altered viscosity or diffusional turnover. Consistent with this hypothesis, microrheology of microspheres in the NTD or R188 mutants showed they have significantly lower viscosity comparative to wild type (**Figs 5B** and **S3G**). FRAP of NT, CT, and R188 also revealed increased exchange rates at centrosomes relative to wild-type protein (**Fig 5C**).

mScarlet-I-cNap1 forms supramolecular assemblies that coalesce (**Figs 1–3**), suggesting that cNap1 itself possesses cohesive properties that promote organelle cohesion. Such a model theorises that separate cNap1 pools coalesce to maintain spatial proximity (**Fig 5D**). Since rootlets are already known to promote centrosome cohesion [3], this hypothesis was tested by targeting cNap1 ectopically to cellular structures not containing rootlets. Three different mScarlet-I-cNap1 fusion proteins were created, targeting mScarlet-I-cNap1 to lysosomes, the Golgi, and mitochondria. These constructs are termed lyso-cNap1, Golgi-cNap1, and mito-cNap1 (see **Materials and methods** for details). Note that cNap1 has not been reported to localise to these organelles. Lyso-cNap1, Golgi-cNap1, and mito-cNap1 had different shapes related to the structure and dynamics of the targeted organelles. Lyso-cNap1 formed spherical shells surrounding lysosomes marked by LysoTracker (**Fig 5E**). Mito-cNap1 and Golgi-cNap1 formed adjacent to or coincident with either mitochondria or the Golgi, respectively (**Fig 5F** and **5G**), demonstrating that the shape of cNap1 assemblies changes when targeted to different organelles, forming an external coating in each case.

Targeting cNap1 to either lysosomes, the Golgi or the mitochondria promoted organelle cohesion in all cases. Whereas in control or untransfected cells, LysoTracker positive vesicles were spaced out within the cytoplasm, lyso-cNap1 coated lysosomes grouped together, frequently forming groups cohered in a honeycomb shape, an arrangement not seen in wild-type cells (**Fig 5E** and **5H**). Time-lapse imaging revealed that lyso-cNap1 coated lysosomes had reduced movements relative to controls, over both minutes (**S3H Fig)** and seconds timescales (**S9 and S10 Movies**). Similarly, mito-cNap1 coated mitochondria cohered together in groups with altered spatial arrangement and reduced movement relative to untransfected controls, visible in live-cell time-lapse imaging (**S11 and S12 Movies**) and fixed-cell imaging (**S3I Fig)**.

Given that these organelles do not contain rootlets, this suggests that cNap1 itself can mediate organelle cohesion, and indeed rootlet fibres were not detected after co-staining for rootletin (**S3J Fig**). To test whether cNap1 condensates are also sufficient to promote centrosome cohesion, I expressed mScarlet-I-cNap1 in U2OS cells, which maintain high levels of centrosome cohesion [23,29], that can be reduced by siRNA-mediated knockdown of rootletin with a previously described siRNA [3]. cNap1-mScarlet-I expression significantly increased centrosome cohesion in rootletin siRNA-treated cells (**Fig 5I**). Together, these results show that cNap1 promotes centrosome cohesion in the absence of rootlets.

## Discussion

The precise mechanisms of centrosome cohesion are unclear, despite the identification of key molecular players [3,26]. Current models of centrosome cohesion indicate that rootlet fibres entangle to link centrioles [3,9,18,23], opposing cytoskeleton-generated forces [20,22,41]. Collectively, the results in this study provoke the hypothesis that cNap1 also directly forms inter-centriolar linkages that maintain centrosome cohesion (**Fig 5J**). Proximal centrioles form dynamic contact sites that are directly bridged on the nanoscale by cNap1 (**Figs 1** and **2**). cNap1 accumulates at micromolar concentrations on centriole termini and forms viscous supramolecular assemblies that promote organelle cohesion, even in the absence of rootlets (**Figs 3** and **5**). This model may explain previous observations that proteins with no known role in rootlet formation are required for centrosome cohesion [17].

Previous electron microscopy data in bovine thymocytes described an “amorphous density” or “hinge” between proximal centriole pairs and partially inside the centriolar lumen [42,43], supporting the observations by U-ExM in this work. Super-resolution light microscopy has also described cNap1 as the most distal proximal centriole component [44]. There is significant flexibility in the maintenance of centrosome cohesion, with centrioles able to transiently separate [23,24]. It is possible that viscous cNap1 material properties allow organelle plasticity to be balanced against solidity in response to physical force from the cytoskeleton. Future work will be required to clarify the precise material properties of cNap1, to understand whether it is viscoelastic over timescales not probed in this study, for example, with active rheology of purified components. Previous work has not included purification of full-length cNap1 or rootletin, motivating the use of parallel in-cell approaches to measure cNap1 material properties.

cNap1 anchors or nucleates rootlets at centrioles, since cNap1 disruption prevents rootlet formation at centrosomes [3,9,26,29]. Consistent with these observations, cNap1 binds specifically to rootletin fibre termini (**Figs 1** and **4**). The data here therefore suggest a dual model of cNap1 function, both anchoring rootletin fibres as 2 foci, and directly promoting centrosome cohesion through coalescence of the foci (**Fig 5J**). One interpretation is that cNap1 condensates create a phase-separated environment that promotes rootlet fibre anchoring or nucleation at the proximal centriole. This model is reminiscent of others proposed for the pericentriolar material—a different centriole protein coat that has also been suggested to phase separate to nucleate microtubules [45,46]. cNap1 has extensive intrinsically disordered regions according to PONDR (**S3K Fig**.) [47], supporting the possibility that it could phase separate through weak interactions between these domains. However, future work will be required to understand in more detail how cNap1 assembles in protein copy numbers of a few hundred at the proximal centriole to nucleate rootlets, since it is alternatively possible that it assembles using site-specific protein–protein interactions unrelated to phase separation.

Overall charge has been suggested to regulate cNap1 oligomerisation, through multisite phosphorylation from the kinase Nek2 [34]. Since multivalent charge–charge interactions are known to regulate condensate formation [48], an interesting future direction of investigation could be to determine whether phosphorylation-dependent cNap1 condensate formation and rootlet end-binding control its centrosomal functions.

In contrast to membranous organelles, less is known about how non-membrane-bound organelle fusion and fission is maintained. cNap1 intra-organelle assemblies at centriole contacts have a number of the characteristics that are used to define membrane–membrane contact sites [49], including dedicated tethering machinery that does not induce full fusion of the rest of the organelle. In this regard, it is interesting to note that most membrane contact sites are maintained by multiple tethering complexes [50], a feature apparently shared by centrosomes that have both rootletin and cNap1-based tethering.

The data here provide a framework to understand the effects of cNap1 disease-causing mutations in the future. One possibility is that disease-causing mutations alter the material properties of cNap1 molecular assemblies. The cNap1 R188 truncation disrupts centrosome cohesion as a dominant negative and forms cytoplasmic condensates with reduced viscosity and altered molecular mobility (**Figs 4** and **5**), illustrating how the effects of other mutations may be rationalised in future work. However, it should be noted that unidentified protein–protein interactions may also be the primary source of disruption in truncated cNap1 mutants.

cNap1 is not conserved throughout the Animalia kingdom, in contrast to its paralog rootletin [5]. The organelle paralogy hypothesis suggests that paralogous duplication is a mechanism for the diversification of membrane-bound organellar function during evolution [51]. This suggests the untested hypothesis that cNap1 has evolved to impart additional centrosomal functionality to mammals.

In conclusion, this work suggests a model of centrosome cohesion using dynamic cNap1 assemblies that form an intra-organelle contact site. More generally, this provides insight into how a non-membrane-bound organelle forms organelle–organelle contacts within the cellular interior.

## Materials and methods

### Cell culture and chemicals

Human U2OS cells were obtained directly from the American Type Culture Collection (ATCC HTB-96). U2OS and HeLa Kyoto cell lines were grown in Dulbecco’s modified Eagle’s Medium (DMEM) supplemented with 10% fetal calf serum, Glutamax, and 100 μg/ml penicillin/streptomycin and maintained at 37°C with 5% CO_2_. hTERT-HPNE human cells were grown in a 1:1 mixture of M3 Base F medium (Incell Corp) and DMEM, with 5% fetal calf serum, 2 mM glutamine, 10 ng/ml EGF, and 750 ng/ml puromycin. Cilia were induced in hTERT-HPNE by 24 h growth in complete medium with 0.2% fetal calf serum. Cell lines were confirmed as mycoplasma free. All tissue culture reagents were purchased from Sigma-Aldrich unless otherwise stated. DNA transfection was with lipofectamine 3000 (Invitrogen) or jetPRIME (Polyplus) according to the manufacturer’s instructions.

### CRISPR/cas9-mediated genome editing

Endogenously tagged cNap1-mScarlet-I and rootletin-meGFP (CROCC-meGFP) U2OS cells were produced with the methods described in [52]. Donor plasmids consisted of two 800-bp homology arms surrounding the C-terminus of either the *cNap1* or *CROCC* genomic reference sequence. These arms were inserted into plasmids such that they flank mScarlet-I or meGFP coding sequence. The *cNap1* donor plasmid was purchased from Thermo Fisher GeneArt. The *CROCC* donor plasmid was constructed by In-fusion cloning (see molecular cloning for In-fusion methods).

sgRNA sequences were designed in Benchling software, selecting optimal on and off target activity as close to the target site as possible. Guide RNAs did not target the donor plasmid. Guide RNA sequences (5′—3′) for *cNap1* were: TCCAGGTAGCAGCCACAGCC (Strand 1), CTGTGGCTGCTACCTGGAGG (Strand -1), TCCTGGCTGTGGCTGCTACC (Strand -1). Guide RNA sequences (5′—3′) for *CROCC* against the +ve strand were as follows (5′—3′): CCAGCAGGAGCTCATTTCTC, CCAGAGAAATGAGCTCCTGC, and CAGGAGCTCATTTCTCTGGG.

Guide RNA sequences were cloned into pSpCas9(BB)-2A-Puro (PX459) (Addgene plasmid 48139) for expression. Guide RNA and the donor plasmid were co-transfected. After a week, single cells were sorted for mScarlet-I or meGFP positivity and grown as clones for PCR screening. Genomic insertion of fluorescent proteins was screened by overlapping genomic PCR. PCR primers were designed either side of the insertion site in clone manager suite, ensuring no false priming. PCR primer annealing temperature was optimised across a temperature gradient before screening clones. For CROCC-meGFP, primer sequences were as follows 5′-3′: GGCTTGGATCTAAGGAGG and GGCTGGCCTTACCTTCCCTT. For cNap1-mScarlet-I primer sequences were as follows 5′-3′: GATTCGTGTATGTGGTAGAG and CTATCACAGTGCATGGTGTA. Tag insertion was detected based on PCR product size, approximately 700 bps larger with fluorescent protein insertion. PCR for screening was with DREAMtaq (Invitrogen) according to the manufacturer’s instructions, run with Hyperladder 1 DNA marker (Bioline). Selected clones were confirmed to have centrosomal localisation as expected by Airyscan confocal imaging, concurring with previously reported antibody staining. Cell lines were also validated by removing fluorescent signal using siRNA-mediated knockdown of either cNap1 or rootletin, using the methods described in the siRNA methods section.

### Airyscan and confocal imaging

All images except those in **S3A Fig** were acquired on a Carl Zeiss LSM 880 Airyscan confocal laser scanning microscope, controlled by Zen Black software. Lenses used were 100× NA 1.46 oil, 63× NA 1.4 oil, 40× NA 1.3 oil, and 40× NA 1.2 water immersion objectives, with optimised cover glass correction where possible. Airyscan images were acquired in SR mode and processing was performed with automatic settings in Zen Black. Pixel size and Z-slice size were optimised, depending on the scan area of each experiment (which was variable), using the optimal function in Zen. Other imaging parameters including scan speed and image averaging were variable for each experiment, but did not change between comparative samples. Laser power was adjusted to minimise bleaching and cellular toxicity in live-cell experiments. Detector gain was adjusted to ensure pixel intensities were never saturated or clipped. The median (± median absolute deviation) lateral and axial resolution of the system was measured at 198 ± 7.5 nm and 913 ± 50 nm (full width at half-maximum), respectively, through imaging of a sub resolution fluorescent bead. Brightness and contrast were adjusted on images for display purposes, but never unequally between comparative samples.

### Live-cell time-lapse imaging and FRAP

Cells were imaged in L15 CO_2_-independent medium at 37°C. Cell health in the conditions used was optimal since cell growth continued. For live-cell time-lapse, image size and frequency of acquisition varied depending on the timescale of events observed and scan area. For high-resolution imaging of centrosomes, a 100× NA 1.46 oil lens was used. Autofocus was at every time point using the Zeiss definite focus autofocus system. A stage top piezo was used for high-speed z-stack imaging. FRAP was performed essentially as described previously [23]. Selected image regions were bleached with a 561-laser line at 100% for the minimum time required to cause approximately 50% fluorescence loss. The bleach duration was constant in all samples. Cells were imaged in a single z-plane following bleaching, at approximately 0.7 s intervals. Analysis was in Microsoft Excel and GraphPad Prism. Images were background subtracted and data was normalised by taking the minimum and maximum values as 0 and 100%, respectively. For each experiment, a mean recovery curve from multiple cells was calculated. With the imaging conditions used, bleaching was minimal, as determined by measuring the change in intensity after running the experiment with identical settings except for the FRAP bleach.

### Expansion microscopy

U-ExM was as described in [31,32,53]. Cells were seeded on 12-mm coverslips overnight, before fixation for 5 h in humid conditions at 37°C in 1.4%/2% formaldehyde (F8775 Sigma)/acrylamide (A4058 Sigma). Gelation was in U-ExM monomer solution, consisting of 23% w/v sodium acrylate (408220 Sigma), 10% w/v acrylamide, and 0.1% w/v N,N′-methylenbisacylamide in PBS. Approximately 0.5% tetramethylethylenediamine (17919, Thermo Fisher Scientific) and 0.5% ammonium persulfate (17874, Thermo Fisher Scientific) were added to the monomer solution directly prior to gelation, with the samples on ice. Gelation was for 5 min on ice and 1 h at 37°C, in a humid chamber. Denaturation was in U-ExM denaturation buffer (200 mM SDS, 200 mM NaCl, 50 mM Tris-BASE in ddH_2_O) at 95°C for 90 min [53]. Gels were expanded overnight at room temperature in ddH_2_O prior to and post antibody incubation. Primary antibody labelling was either overnight at 4°C or 3 h at 37°C in 2% BSA-PBS at 1:250 dilution. Secondary antibody labelling was at 37°C in 2% BSA-PBS at 1:500 dilution for 2.5 h. Gels were washed with PBS 0.1% triton-X after both antibody staining steps. Nuclei were labelled with Hoechst 33342 dye in the final wash step. Gels were mounted in Ibidi μ-slide 2-well glass bottom #1.5 dishes (80286), which were pre-treated in poly-L-lysine or poly-D-lysine and imaged using Airyscan imaging. Primary antibodies used were mouse anti-acetylated tubulin (Sigma Aldrich, T7451), rabbit anti-rootletin (Novus Biologicals, NBP1-80820), rabbit anti-cNap1 (Proteintech, 14498-1-AP), mouse anti-γtubulin (GTU-88, Abcam). For anti-γtubulin only, cells were fixed in 100% ice-cold methanol for 5 min prior to formaldehyde fixation. Secondary antibodies were Alexa 488, Alexa 568, or Atto 565 conjugates. The expansion factor was calculated from measurements of the gel diameter pre and post expansion, and from centriole size in final images when stained with anti-acetylated tubulin, giving values around 4.2 to 4.6. Calculation of the percentage of cells with cNap1 bridges between centrioles was done from 3 independent experiments, measuring a total of approximately 80 cells.

### Antibody validation

cNap1 antibody was validated specifically in U-ExM by confirming that signal was removed by siRNA targeting cNap1 in comparison to non-targeting control siRNA (**S2A Fig**). In standard immunofluorescence, anti-cNap1 staining closely matched cNap1-mScarlet-I fluorescent protein.

### Automated image acquisition and analysis

**S3A Fig** was acquired on a Molecular Devices ImageXpress Micro Confocal. Objective cover glass corrections were optimised to scan with Ibidi μ-slide 8-well dishes and a 40× air objective. Multiple z-sections were obtained and then projected using the Molecular Devices “best” function. Images were analysed in Ilastik and CellProfiler software, using custom-made pipelines. Briefly, cells and cNap1 foci were automatically segmented using pixel-based image classification. Segmented images were further analysed in CellProfiler, using the relate function and to associate cNap1 and cells and therefore count number per cell. Segmented shape parameters were calculated, dividing the major and minor axis lengths to obtain the aspect ratio.

### Molecular cloning

DNA constructs were made by In-fusion HD cloning (Clontech) into the vector pcDNA 3.1, according to the manufacturer’s instructions. Briefly, primers containing complementary 15 bp extensions were designed in the TaKaRa Bio In-fusion online design tool. Both the vector and inserts were amplified by PCR with CloneAmp DNA polymerase. Amplified DNA length was verified by agarose gel electrophoresis. In-Fusion ligation was performed on gel extracted DNA using In-Fusion HD enzyme premix in a total volume of 5 μl at 50°C for 15 min. Clones were screened by Sanger DNA sequencing, restriction digest and microscopy after transfection into mammalian cells. The original cNap1 cDNA template was provided by Andrew Fry (University of Leicester, United Kingdom).

### Design and imaging of mito-cNap1, lyso-cNap1, Golgi-cNap1, and cNap1 truncation constructs

Mito-cNap1 consists of an N-terminal fusion of a pair of mitochondria targeting sequences from cytochrome c oxidase subunit VIII (COX8) [54], separated by a short linker, to give the following amino acids: MSVLTPLLLRGLTGSARRLPVPRAKIHSLPPEGKLGMSVLTPLLLRGLTGSARRLPVPRA. This was fused in frame to mScarlet-I-cNap1, therefore forming COX8-mScarlet-I-cNap1. Lyso-cNap1 consists of cNap1-mScarlet-I fused in frame to human lysosomal-associated membrane protein 1 (*LAMP-1*) [55], to create LAMP1-cNap1-mScarlet-I. A negative control consisted of LAMP1-mScarlet-I. Golgi-cNap1 consists of a C-terminal fusion of a GRIP domain [56], consisting of the C-terminal 98 amino acids of Golgin-245, to cNap1-mScarlet-I. This forms cNap1-mScarlet-I-GRIP. A negative control consisted of mScarlet-I-GRIP. cNap1 truncations were made by HD In-fusion cloning from the full-length gene. Constructs were imaged approximately 18 h after transient transfection with Lipofectamine 3000. A dose-dependent increase in construct expression level was observed over time, generally resulting in gradual coating of the exterior of the targeted organelle. Live-cell imaging of mito-cNap1 or lyso-cNap1 dynamics was after preincubation in 0.5μg/ml Hoechst 33342 for 30 s before incubation in fresh imaging medium. Lysosome size was measured with custom made Ilastik and CellProfiler software pipelines.

### siRNA transfection

siRNA targeting rootletin/CROCC (gene name *CROCC*) was as previously described by [3] and is as follows: 5′-AAGCCAGTCTAGACAAGGA-3′. This siRNA notably has a strong centrosome splitting phenotype relative to other rootletin-targeting siRNA [3] and was custom synthesised by Horizon Discovery. Non-targeting negative control siRNA and siRNA targeting cNap1 were ON-TARGET *plus* pools from Horizon Discovery (D-001810-OX and L-012364, respectively). siRNA transfection was with RNAiMAX (Thermo Fisher Scientific), following the manufacturer’s instructions. Briefly, for 96-well transfections, cells were transfection with 25 nm of siRNA, and 0.25 μl lipofectamine per well. Cells were analysed either 48 or 72 h after transfection. The efficacy of both cNap1 and rootletin siRNA knockdown was confirmed by loss of fluorescence in cNap1-mScarlet-I and rootletin-meGFP cells.

### Standard immunofluorescence and dye staining

Cells were fixed in either 4% paraformaldehyde in PBS pH 7.4 for 15 min or ice-cold 100% methanol for 5 min. Fixatives were freshly prepared. Paraformaldehyde was quenched in 0.1 M NH_4_Cl in PBS (pH 7.4). Cells were permeabilised in 0.1% Triton in PBS and blocked in 3% bovine serum albumen (Thermo Fisher Scientific) in PBS. Antibodies used were: mouse anti-gamma tubulin GTU-88 (1:1,000 Sigma-Aldrich T6557), rabbit anti-GM130 (1:1,000 Abcam, ab52649), rabbit anti-PCNT (1:1,000 Abcam ab4448), and rabbit anti-CEP135 (1:1,000 Abcam ab75005). MitoTracker deep red (Thermo Fisher Scientific) was incubated at culture conditions at 100 nm for 30 s before replacing with fresh medium for imaging. LysoTracker was used at 75 nm, added directly prior to imaging, and kept in the imaging medium.

### FCS-calibrated imaging

FCS-calibrated imaging was performed as described in [52,57], to measure fluorescent protein concentration. Cells were seeded into 8-well Ibidi dishes the day prior to imaging and changed into prewarmed L15 media on the day of imaging. FCS and imaging was performed on a Zeiss 880 ConfoCor microscope with 40× 1.2 NA water immersion objective, incubated at 37°C. For FCS measurements, samples were excited with the 561-nm laser line, using minimal power to minimise bleaching, cellular toxicity, and photophysical effects. The pinhole was set to 1 airy unit and light was detected with a GaAsP detector at 605 to 676 nm. A cover glass correction was performed prior to each experiment to account for variations in dish thickness. Thus, the counts per molecule of Alexa-568 was maximised by adjusting the lens correction collar, using the same sample dish already containing cells, but in a separate well.

Approximately 100 nm Alexa 568 dye in water was used to measure the size of the confocal volume (see FCS analysis section), approximately 20 μm above the coverslip. Cellular FCS measurements were taken with four 10-s readings in the same position, in the cytoplasm away from any discernible structure. Wild-type U2OS cells without any fluorescent fusion protein were measured to determine the background fluorescence count rate in FCS. To make a calibration line for FCS-calibrated imaging, 12 cells overexpressing mScarlet-I-cNap1 were measured in the cytoplasm, taking care to avoid photobleaching. Cells were measured with both FCS and imaging (as below). Subsequent imaging of further transiently transfected mScarlet-I-cNap1 samples or endogenously tagged samples were with identical imaging settings.

Imaging was in standard confocal mode (not Airyscan), using the same water immersion lens, laser power and pinhole diameter settings used for the FCS. Since cytoplasmic concentration was of primary interest in overexpressing cells, imaging settings were optimised for the cytoplasm (rather than for cNap1 cytoplasmic condensates), with pixel size and dwell time optimised for the dimmest sample. Image x, y, and z pixel size was 100 and 400 nm, respectively. In calibration cells, images were taken before FCS measurements to minimise the effect of photobleaching from FCS.

### FCS analysis

#### Dye calibration

FCS data analysis was performed in Fluctuation Analyzer software [57], to calculate autocorrelation functions, correct for background and bleaching, and perform fits. The autocorrelation function is: $G(\tau )=\frac{<\delta I(t)*\delta I(t+\tau )>}{{<I>}^{2}}$, where *I* is the intensity and <> time averaging. The “base frequency” was set to 1,000,000 in Fluctuation Analyzer. Dye calibration measurements were fit to a 1-component model of diffusion with triplet-like blinking: $G(\tau )={\frac{\left(1+\frac{\theta_{T}}{1-\theta_{T}}\exp\left(-\frac{\tau }{\tau_{T}}\right)\right)}{N}(1+(\frac{\tau }{\tau D}))}^{-1}{\left(1+k^{-2}(\frac{\tau }{\tau D})\right)}^{-0.5}$ to obtain the diffusion time τD and structural parameter *k*. N is the apparent number of molecules in the confocal volume, ɵ_T_ is the fraction of molecules in a nonfluorescent state, and τ_T_ their lifetime. The width of the confocal volume was calculated using: $w_{0}=2\sqrt{D_{dye}\tau_{D}}$, where D_dye_ is the diffusion coefficient of the dye, and 521.46 μm^2^/s was used. This method gave a value of approximately 225 nm for the confocal volume lateral waist size. The confocal volume was calculated with: $V=\pi^{3/2}{w_{0}}^{3}k$, where V is the effective confocal volume and k is the ratio of the axial to lateral radius of this volume (estimated from autocorrelation fitting). Concentration was calculated using: $C=\frac{N}{VN_{A}}$, where N_A_ is the Avogadro constant.

#### Cell measurements

Data was discarded if it showed significant bleaching or cell movements, visible in the count rate. The “base frequency” was set to 100,000 in Fluctuation Analyzer to calculate the autocorrelation function, and the offset was determined from measurements in wild-type cells not expressing fluorescent protein. To obtain the number of particles in the effective confocal volume (N), autocorrelation functions were fitted with a 1-component model of anomalous diffusion with fluorescent protein blinking: $G(\tau )=\frac{\left(1-\theta_{\tau }+\theta_{\tau }exp(-\frac{\tau }{\tau_{T}})\right)}{N}{\sum_{i=1,2}f_{i}(1+{\left(\frac{\tau }{\tau D}\right)}^{\alpha })}^{-1}{\left(1+k^{-2}{\left(\frac{\tau }{\tau D}\right)}^{\alpha }\right)}^{-0.5}$. To compute fluorophore concentration, the number of molecules N obtained from the fit was divided by the effective confocal volume (calculated from dye calibration measurements).

An experiment-specific conversion factor was used to convert relative fluorescence intensity measurements into absolute concentrations, by using images and FCS measurements taken in the same cells. The mean intensity of a 6 × 6 voxel area was taken centred on the FCS measurement position. Plotting image intensity versus absolute concentration in the cytoplasm from a population of cells gave a straight line from which the conversion factor was obtained, indicating use of the microscope in the linear range when correlating FCS and imaging measurements.

Concentrations were converted into number of molecules per pixel using: $N=N_{A}C\;\Delta p$, where N is the number of particles in the pixel, N_A_ is the Avogadro constant, and Δp is pixel volume in x, y, and z. The number of cNap1 proteins per centrosome was calculated as the product of this N and cNap1 centriolar size. Centriolar cNap1 size was calculated from the number of manually segmented cNap1 positive pixels in each single confocal image. For each cell, the mean of both centrioles was calculated. This measure of centriolar size agreed well with an estimate from the expansion microscopy shown in **Fig 2**. Intensities at centrosomes were taken by manual segmentation to acquire the mean pixel intensity.

### Mean square displacement analysis

Cells were seeded overnight in μ-slide 8-well dishes (Ibidi, 80826). The next day, they were transfected with mScarlet-I-cNap1 cDNA using lipofectamine 3000, and simultaneously incubated with green fluorescent microspheres (PS Speck P7220 Thermo Fisher Scientific), at a bead concentration of 2.25 × 10^6^/ml. After 18 h, this resulted in a population of cells in which beads were encapsulated inside cNap1 condensates. Medium was removed, washed twice with warm media, and then replaced with fresh, CO_2_-independent Leibovitz’s L-15 medium imaging medium. Cells without fluorescent bead encapsulation were excluded from the analysis. Time-lapse imaging of bead movement was with a Zeiss LSM 880 confocal laser scanning microscope operating in standard confocal mode with a 100× NA 1.46 oil immersion objective. The time interval was 10 s between frames, acquiring images with xy scaling of 0.082 μm and 0.393 μm z-scaling. A single z-slice was acquired. Two colour images were acquired, with filters 493 to 594 and 599 to 696 nm, to acquire images of both microspheres and mScarlet-I, respectively. Shorter time interval imaging (<1 s intervals) was also tested and resulted in a similar diffusion coefficient. The CTD truncation formed very few condensates which did not encapsulate beads, and hence was not measured. Tracks were acquired in 3 independent experiments for wild-type cells and 2 independent experiments for NTD and R188 mutants. Cytoplasmic measurements were obtained in untransfected cells. These samples occasionally showed beads with rapid directional movement rather than diffusion, and these beads were not included in the analysis.

Single-particle tracking was performed with the FiJi Trackmate plugin [58], using the LoG detector and simple LAP tracker. The automatic spot detection and track linking was manually checked in each frame for accuracy, and tracks with fewer than 60 consecutively tracked spots were discarded. For each track, mean square displacement analysis was performed using the MATLAB class MSD Analyzer [38]. The MSD is $MSD(t)=<[{x(t+\tau )-x(t)]}^{2}>+{y(t+\tau )-y(t)]}^{2}>$, τ is the time delay and x(t) and y(t) are the coordinates of a particle at time t. MSD = 4Dt^α^, where D is the particle diffusion coefficient and α is the diffusive exponent. α and D were obtained from fits in MSD Analyzer, and tracks were corrected for drift as described [38,59]. D was obtained from the first 25% of the data, to account for the decreasing reliability of longer time delays, using a straight line linear weighted fit of the MSD in MSD Analyzer. Viscosity values were calculated through the Stokes–Einstein equation, =kBT/6πDR, where kB is Boltzmann’s constant, T is temperature, and R is particle radius. The noise floor of the measurements was 0.0000015 μm^2^/s, calculated from immobile beads dried to the bottom of a well.

### Protein disorder analysis

Intrinsically disordered regions in cNap1 were predicted using the Predictor of Naturally Disordered Regions (PONDR) algorithm [60].

### Statistical analysis

Statistical analyses and graphical representations were in GraphPad Prism 5 software. Statistical tests are listed in the figure legends.

## Supporting information

S1 Fig Construction and validation of endogenously tagged cNap1-mScarlet-I and CROCC-meGFP in U2OS cells. (**A**) Junction PCR screening of genomic DNA for insertion of mScarlet-I at the C-terminus of *cNap1*. Clone 7 was selected since it is homozygous for cNap1-mScarlet-I. The selected clone is indicated by *. The DNA ladder is Hyperladder 1 from Bioline. (**B**) Junction PCR of genomic DNA, screening for insertion of mScarlet-I at the C-terminus of *cNap1*. This shows a comparison of clone 7 with a heterozygous pool. Lanes are loaded in triplicate to exclude the possibility of lane-to-lane variability. (**C**) Junction PCR of genomic DNA, screening for insertion of meGFP at the C-terminus of *CROCC*. This shows a heterozygous clone. (**D**) Centrosome cohesion in wild type and cNap1-mScarlet-I/rootletin-meGFP cells, assessed by immunofluorescent staining of centrosomes with anti-PCNT antibody in a population of cells. Centrosomes were classed as split if 2 PCNT positive foci were present and separated by more than 1.6 μm, measuring in 240 and 329 wild-type and genome-edited cells, respectively. The images show maximum intensity projections of confocal Airyscan z-stacks. Scale: 10 μm. (**E**) Endogenously tagged cNap1-mScarlet-I co-stained with anti-CEP135. Scale: 1 μm. (**F**) Distance between the centroids of cNap1-mScarlet-I foci of the cell shown in **Fig 1B** during time-lapse imaging. (**G**) Distance between the centroids of cNap1-mScarlet-I foci during time-lapse imaging. Each colour plots a different cell. Data underlying this figure can be found in S1 Raw Data.
(PDF)

S2 Fig U-ExM of centrioles and cNap1. **(A)** Validation of anti-cNap1 U-ExM staining with siRNA. U2OS cells were treated with either siRNA targeting cNap1 (left panel), or non-targeting siRNA (right panel), and then processed identically for U-ExM. (**B**) U-ExM expanded U2OS cells stained with anti-cNap1 (grey) and anti-acetylated tubulin (magenta). Each image is a different cell. Maximum intensity z-projections are shown. (**C**) U-ExM expanded hTERT-HPNE cell stained with anti-cNap1 (grey) and anti-acetylated tubulin (magenta). (**D**) U-ExM expanded U2OS cells stained with anti-rootletin (green) and anti-acetylated tubulin (red). Each image is a different cell. Scale: 200 nm throughout.
(PDF)

S3 Fig In vivo behaviour of mScarlet-I-cNap1 condensates. (**A**) cDNA-based cNap1-mScarlet-I expression results in either centrosomal or cytosolic patches (red). Centrosomes are co-stained with gamma-tubulin (green), and centrosome position is indicated with arrows. White lines in the bottom panels denote nuclei. Scale bar: 4 μm. The histogram shows the number of mScarlet-I-cNap1 patches per cell in a population of 388 cells, acquired with automated imaging and analysis as detailed in **Materials and methods**. The xy graph plots mScarlet-I-cNap1 area against aspect ratio (long axis/short axis) in approximately 4,000 patches, where a circle has an aspect ratio of 1. (**B**) mScarlet-I does not form condensates when overexpressed (top panel—red), but cNap1 does (bottom panel—green). (**C**) FCS-calibrated imaging of cDNA-expressed cNap1-mScarlet-I. The dot plot shows the cytoplasmic concentration in cells either with or without cytoplasmic condensates. Each dot is a single cell. The dashed line indicates a concentration of 110 nm. The example image is coloured relative to concentration. (**D**) Live-cell time-lapse imaging of a single cytoplasmic mScarlet-I-cNap1 patch over minutes, showing viscous liquid-like shape changes over time. Scale: 1.5 μm. (**E**) Log–log plot of fluorescent microsphere movement inside cNap1 condensates. The graph plots the mean from 51 tracks. The diffusive exponent alpha has a value of 0.93 + 0.04 (goodness of fit adjusted R2 = 0.98), obtained using the MATLAB class MSD Analyzer [38]. (**F**) Half-bleaching FRAP of an mScarlet-I-cNap1 patch in the cytosol shows limited exchange over approximately 30 s. The bleached region is located at the bottom and images show successive indicated time points. Scale bar: 2 μm. (**G**) Mean squared displacement of fluorescent microspheres diffusing in cNap1 NT, R188, or wild type. The lines show weighted means (±SEM) from *N* = 51 (wild type), *N* = 40 (NT), and *N* = 20 (R188) tracks. (**H**) Right panel: dual colour time-lapse imaging of lyso-cNap1 (red) and LysoTracker (green). Scale: 5 μm. Left panel: dual colour time-lapse imaging of Lamp1-mScarlet-I (red; right panel) and LysoTracker (green). Time-lapse was performed over minutes as indicated. Scale: 5 μm. (**I**) Top panel: immunofluorescent imaging of mito-cNap1 (red), DNA (Hoechst 33342, blue), and MitoTracker (yellow). Bottom panel: immunofluorescent imaging of Golgi-cNap1 (red), DNA (blue), and anti-GM130 (green). The arrows in both panels denote a cell expressing either mito-cNap1 or Golgi-cNap1, respectively, and the stars denote untransfected control cells in the same image. Scale bars: 7 μm and 10 μm in the top and bottom panels, respectively. (**J**) Lyso-cNap1, Golgi-cNap1, and mito-cNap1 do not induce de novo rootlet formation, as detected by anti-CROCC staining. Scale: 5 μm. (**K**) Predicted cNap1 disorder in each residue according to PONDR VL-XT score [47]. A score in the range 0.5–1 indicates possible disorder. Data underlying this figure can be found in S6 Movie and S1 Raw Data.
(PDF)

S1 Raw Images Raw gel images. **(A)** S1A (**B**) S1B (**C**) S1C.
(PDF)

S1 Raw Data Raw numerical values for the figures. (XLSX)

S1 Movie Time-lapse Airyscan imaging of endogenous cNap1-mScarlet-I in U2OS cells at one min intervals, for a total time of 30 min, related to Fig 1B. A single slice of a z-stack is shown. Scale: 1 μm.
(AVI)

S2 Movie Time-lapse Airyscan imaging of endogenous rootletin-meGFP and cNap1-mScarlet-I at 12 s intervals in U2OS cells, related to Fig 1C. A sum projection of a z-stack is shown.
(AVI)

S3 Movie Time-lapse Airyscan imaging of endogenous rootletin-meGFP and cNap1-mScarlet-I at 12 s intervals in U2OS cells, related Fig 1D. A sum projection of a z-stack is shown.
(AVI)

S4 Movie Formation of mScarlet-I-cNap1 condensates in the cytoplasm after transfection. Maximum intensity projections at 30 min time intervals in U2OS cells. Scale: 10 μm.
(AVI)

S5 Movie Coalescence of mScarlet-I-cNap1 cytoplasmic condensates. Time frames are taken at 3-min intervals, related to **Fig 3D**. Scale: 2 μm.
(AVI)

S6 Movie Shape changes of mScarlet-I-cNap1 taken at 30-min intervals in U2OS cells. (AVI)

S7 Movie Time-lapse imaging of eGFP-rootletin and mScarlet-I-cNap1 at 0.5-h intervals. Maximum intensity projections are shown. Scale: 10 μm.
(AVI)

S8 Movie Time-lapse imaging of eGFP-rootletin and mScarlet-I-cNap1 at 5-min intervals. Scale: 2 μm.
(AVI)

S9 Movie Time-lapse Airyscan imaging of LysoTracker (green), lyso-cNap1 (red), and Hoechst 33342 (blue) at 2-s intervals. A single z-slice is shown. Scale: 3 μm.
(AVI)

S10 Movie Time-lapse Airyscan imaging of LysoTracker (green) and Hoechst 33342 (blue) at 2-s intervals in an untransfected U2OS cell. A single z-slice is shown. Scale: 3 μm.
(AVI)

S11 Movie Time-lapse Airyscan imaging of mito-cNap1 (red), MitoTracker (yellow), and Hoechst 33342 (blue) at 2-s intervals in a U2OS cell. A single z-slice is shown. Scale: 3 μm.
(AVI)

S12 Movie Time-lapse Airyscan imaging of MitoTracker (yellow) and Hoechst 33342 (blue) at 2-s intervals in an untransfected U2OS cell. A single z-slice is shown. Scale: 3 μm.
(AVI)

## Abbreviations

FCS: fluorescence correlation spectroscopy
FRAP: fluorescence recovery after photobleaching
MSD: mean squared displacement
PONDR: Predictor of Naturally Disordered Regions
siRNA: small interfering RNA
U-ExM: ultra-expansion microscopy
