# Supplementary figures and images for: cNap1 bridges centriole contact sites to maintain centrosome cohesion
Source: PLoS Biol. 2022 Oct 25;20(10):e3001854. doi: 10.1371/journal.pbio.3001854 (PMC9595518; doi:10.1371/journal.pbio.3001854)

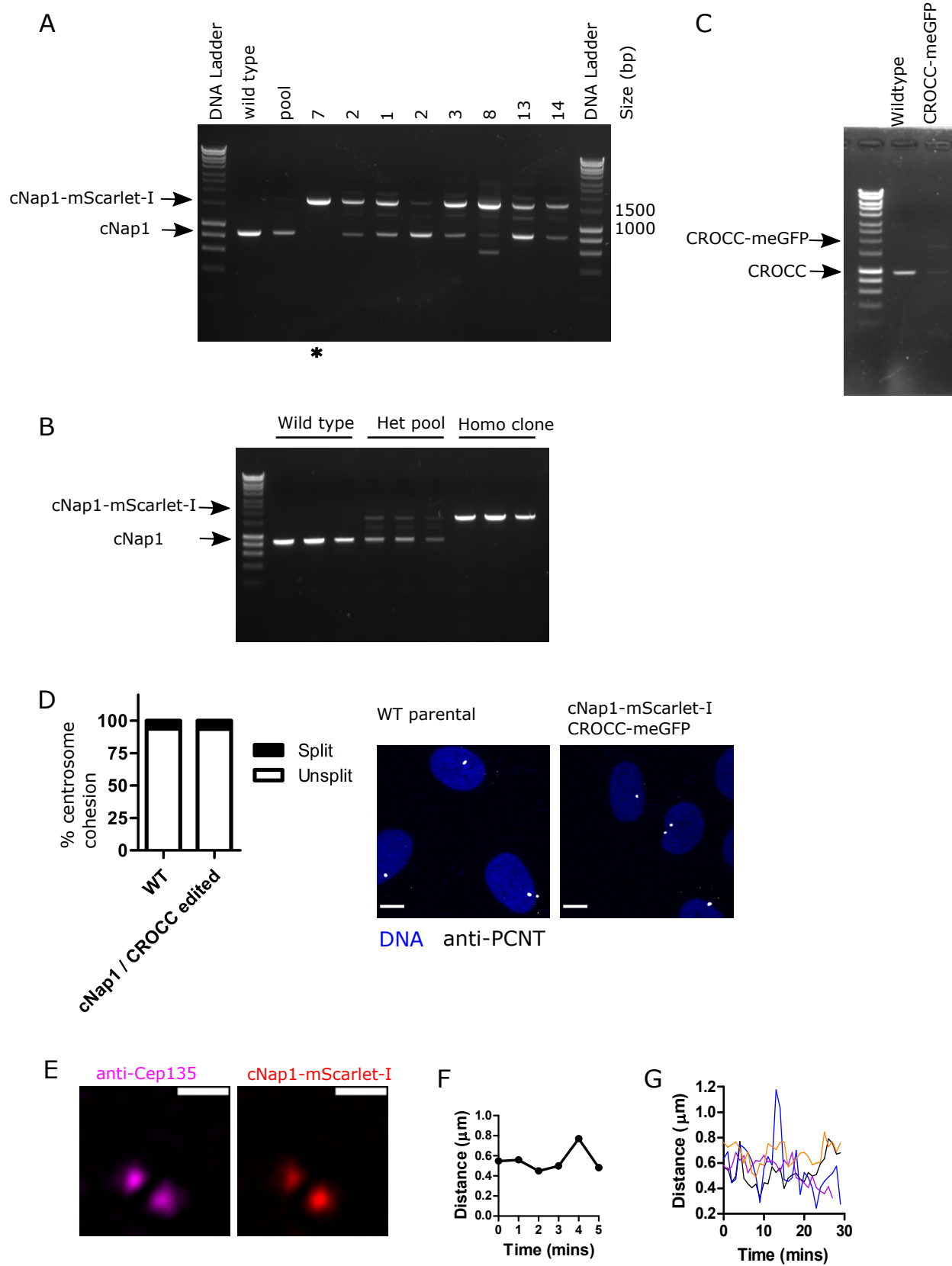

Supplement: S1 Fig — (A) Junction PCR screening of genomic DNA for insertion of mScarlet-I at the C-terminus of cNap1. Clone 7 was selected since it is homozygous for cNap1-mScarlet-I. The selected clone is indicated by *. The DNA ladder is Hyperladder 1 from Bioline. (B) Junction PCR of genomic DNA, screening for insertion of mScarlet-I at the C-terminus of cNap1. This shows a comparison of clone 7 with a heterozygous pool. Lanes are loaded in triplicate to exclude the possibility of lane-to-lane variability. (C) Junction PCR of genomic DNA, screening for insertion of meGFP at the C-terminus of CROCC. This shows a heterozygous clone. (D) Centrosome cohesion in wild type and cNap1-mScarlet-I/rootletin-meGFP cells, assessed by immunofluorescent staining of centrosomes with anti-PCNT antibody in a population of cells. Centrosomes were classed as split if 2 PCNT positive foci were present and separated by more than 1.6 μm, measuring in 240 and 329 wild-type and genome-edited cells, respectively. The images show maximum intensity projections of confocal Airyscan z-stacks. Scale: 10 μm. (E) Endogenously tagged cNap1-mScarlet-I co-stained with anti-CEP135. Scale: 1 μm. (F) Distance between the centroids of cNap1-mScarlet-I foci of the cell shown in Fig 1B during time-lapse imaging. (G) Distance between the centroids of cNap1-mScarlet-I foci during time-lapse imaging. Each colour plots a different cell. Data underlying this figure can be found in S1 Raw Data. (PDF) [file pbio.3001854.s001.pdf]

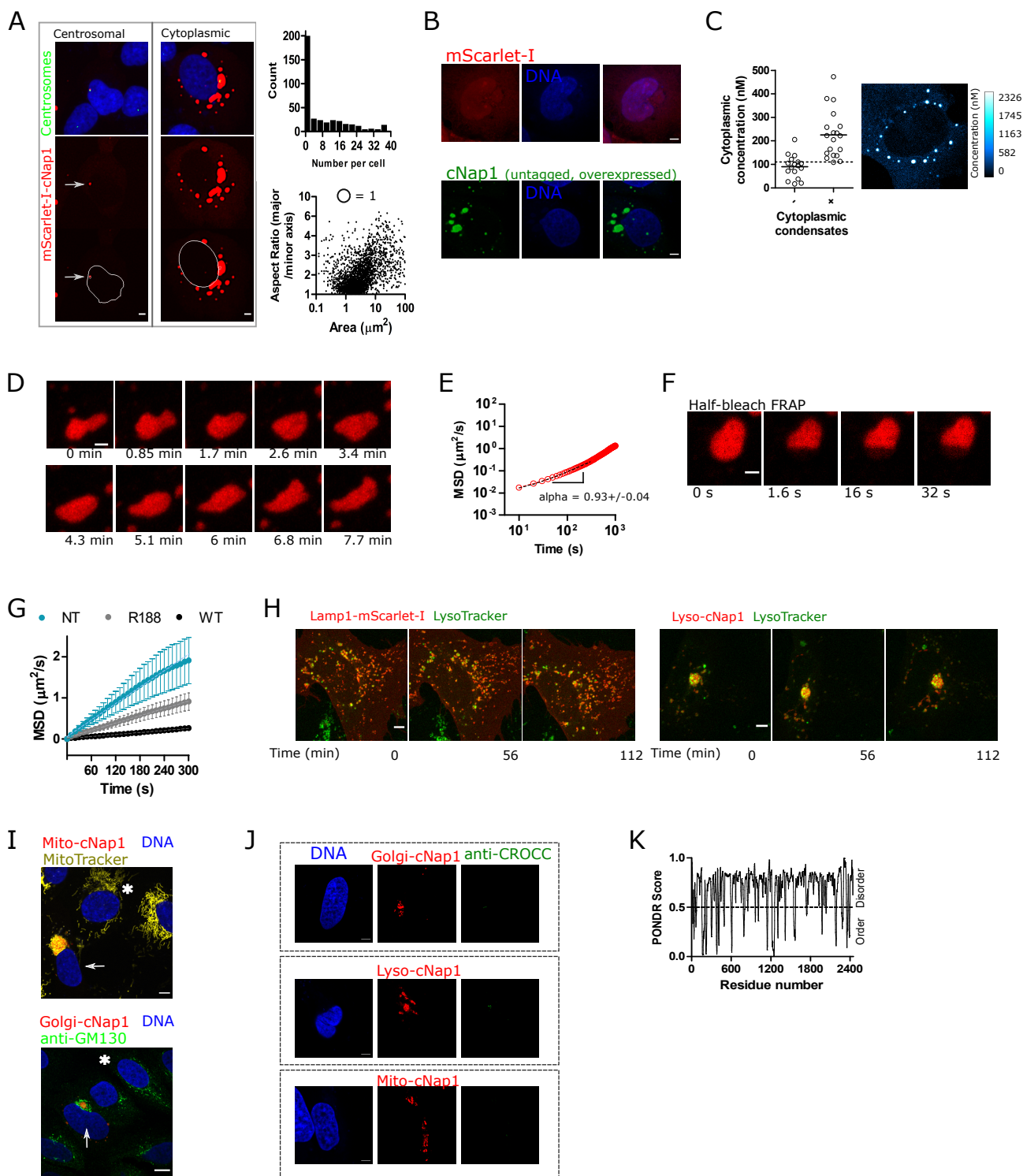

Supplement: S3 Fig — (A) cDNA-based cNap1-mScarlet-I expression results in either centrosomal or cytosolic patches (red). Centrosomes are co-stained with gamma-tubulin (green), and centrosome position is indicated with arrows. White lines in the bottom panels denote nuclei. Scale bar: 4 μm. The histogram shows the number of mScarlet-I-cNap1 patches per cell in a population of 388 cells, acquired with automated imaging and analysis as detailed in Materials and methods. The xy graph plots mScarlet-I-cNap1 area against aspect ratio (long axis/short axis) in approximately 4,000 patches, where a circle has an aspect ratio of 1. (B) mScarlet-I does not form condensates when overexpressed (top panel—red), but cNap1 does (bottom panel—green). (C) FCS-calibrated imaging of cDNA-expressed cNap1-mScarlet-I. The dot plot shows the cytoplasmic concentration in cells either with or without cytoplasmic condensates. Each dot is a single cell. The dashed line indicates a concentration of 110 nm. The example image is coloured relative to concentration. (D) Live-cell time-lapse imaging of a single cytoplasmic mScarlet-I-cNap1 patch over minutes, showing viscous liquid-like shape changes over time. Scale: 1.5 μm. (E) Log–log plot of fluorescent microsphere movement inside cNap1 condensates. The graph plots the mean from 51 tracks. The diffusive exponent alpha has a value of 0.93 + 0.04 (goodness of fit adjusted R2 = 0.98), obtained using the MATLAB class MSD Analyzer [38]. (F) Half-bleaching FRAP of an mScarlet-I-cNap1 patch in the cytosol shows limited exchange over approximately 30 s. The bleached region is located at the bottom and images show successive indicated time points. Scale bar: 2 μm. (G) Mean squared displacement of fluorescent microspheres diffusing in cNap1 NT, R188, or wild type. The lines show weighted means (±SEM) from N = 51 (wild type), N = 40 (NT), and N = 20 (R188) tracks. (H) Right panel: dual colour time-lapse imaging of lyso-cNap1 (red) and LysoTracker (green). Scale: 5 μm. Left pa [file pbio.3001854.s003.pdf]

Original gels

S1A

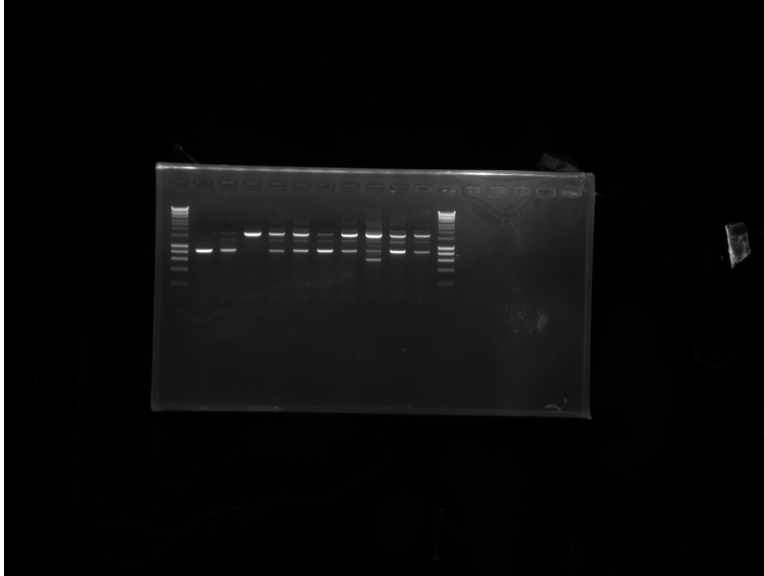

S1B

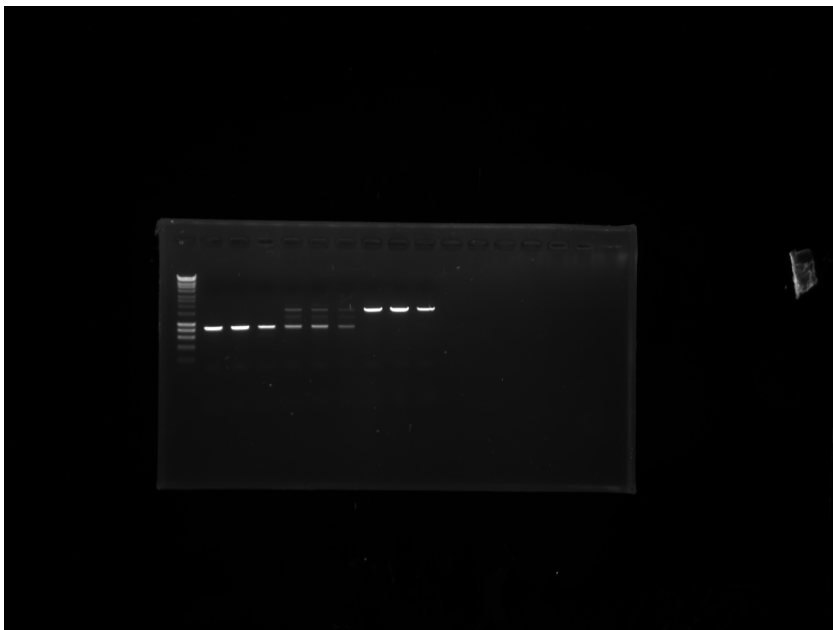

S1C

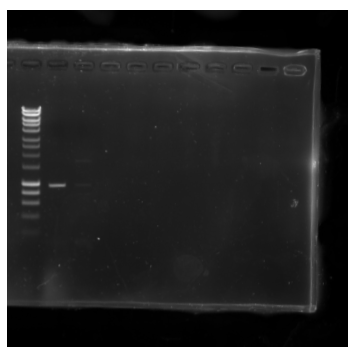

Supplement: S1 Raw Images — (A) S1A (B) S1B (C) S1C. (PDF) [file pbio.3001854.s004.pdf]
