# Supplementary material for: cNap1 bridges centriole contact sites to maintain centrosome cohesion
Source: PLoS Biol. 2022 Oct 25;20(10):e3001854. doi: 10.1371/journal.pbio.3001854 (PMC9595518; doi:10.1371/journal.pbio.3001854)
Supplement: S2 Fig — (A) Validation of anti-cNap1 U-ExM staining with siRNA. U2OS cells were treated with either siRNA targeting cNap1 (left panel), or non-targeting siRNA (right panel), and then processed identically for U-ExM. (B) U-ExM expanded U2OS cells stained with anti-cNap1 (grey) and anti-acetylated tubulin (magenta). Each image is a different cell. Maximum intensity z-projections are shown. (C) U-ExM expanded hTERT-HPNE cell stained with anti-cNap1 (grey) and anti-acetylated tubulin (magenta). (D) U-ExM expanded U2OS cells stained with anti-rootletin (green) and anti-acetylated tubulin (red). Each image is a different cell. Scale: 200 nm throughout. (PDF) [file pbio.3001854.s002.pdf]

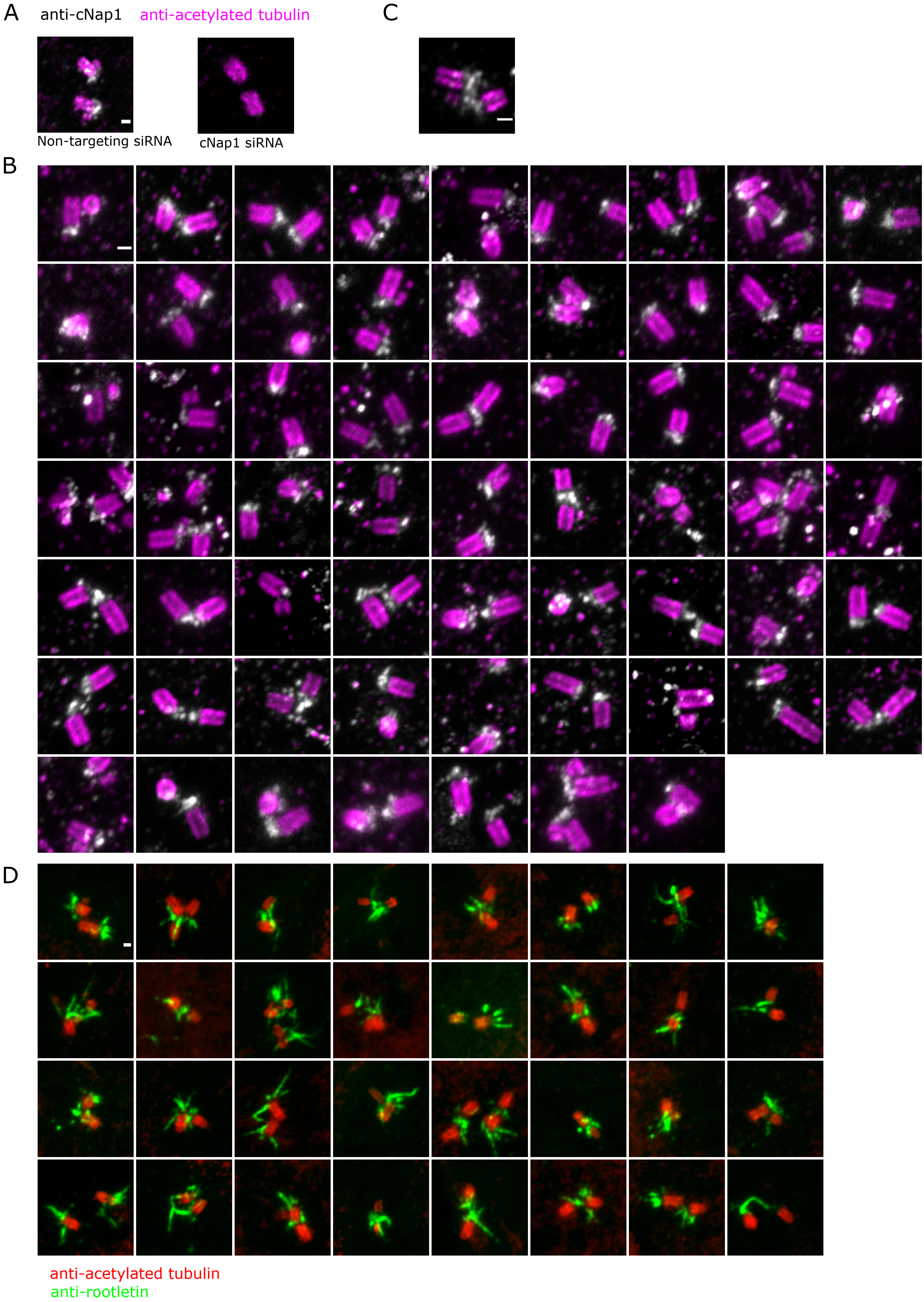
A
anti-cNap1
anti-acetylated tubulin
Non-targeting siRNA
cNap1 siRNA
C
B
D
anti-acetylated tubulin
anti-rootletin
